# Prognostic Value of Complete Resection of the High-Frequency Oscillation Area in Intracranial EEG: A Systematic Review and Meta-Analysis

**DOI:** 10.1212/WNL.0000000000209216

**Published:** 2024-04-01

**Authors:** Ziyi Wang, Jiaojiao Guo, Maryse van 't Klooster, Sem Hoogteijling, Julia Jacobs, Maeike Zijlmans

**Affiliations:** From the Department of Neurology and Neurosurgery (Z.W., J.G., M.v.t.K., S.H., M.Z.), University Medical Center Utrecht Brain Center, University Medical Center Utrecht, Part of ERN EpiCARE, the Netherlands; Department of Pediatrics (J.J.), University of Calgary, Alberta Children's Hospital, Calgary, Canada; and Stichting Epilepsie Instellingen Nederland (SEIN) (M.Z.), Heemstede, the Netherlands.

## Abstract

**Background and Objectives:**

High-frequency oscillations (HFOs; ripples 80–250 Hz; fast ripples [FRs] 250–500 Hz) recorded with intracranial electrodes generated excitement and debate about their potential to localize epileptogenic foci. We performed a systematic review and meta-analysis on the prognostic value of complete resection of the HFOs-area (crHFOs-area) for epilepsy surgical outcome in intracranial EEG (iEEG) accessing multiple subgroups.

**Methods:**

We searched PubMed, Embase, and Web of Science for original research from inception to October 27, 2022. We defined favorable surgical outcome (FSO) as Engel class I, International League Against Epilepsy class 1, or seizure-free status. The prognostic value of crHFOs-area for FSO was assessed by (1) the pooled FSO proportion after crHFOs-area; (2) FSO for crHFOs-area vs without crHFOs-area; and (3) the predictive performance. We defined high combined prognostic value as FSO proportion >80% + FSO crHFOs-area >without crHFOs-area + area under the curve (AUC) >0.75 and examined this for the clinical subgroups (study design, age, diagnostic type, HFOs-identification method, HFOs-rate thresholding, and iEEG state). Temporal lobe epilepsy (TLE) was compared with extra-TLE through dichotomous variable analysis. Individual patient analysis was performed for sex, affected hemisphere, MRI findings, surgery location, and pathology.

**Results:**

Of 1,387 studies screened, 31 studies (703 patients) met our eligibility criteria. Twenty-seven studies (602 patients) analyzed FRs and 20 studies (424 patients) ripples. Pooled FSO proportion after crHFOs-area was 81% (95% CI 76%–86%) for FRs and 82% (73%–89%) for ripples. Patients with crHFOs-area achieved more often FSO than those without crHFOs-area (FRs odds ratio [OR] 6.38, 4.03–10.09, *p* < 0.001; ripples 4.04, 2.32–7.04, *p* < 0.001). The pooled AUCs were 0.81 (0.77–0.84) for FRs and 0.76 (0.72–0.79) for ripples. Combined prognostic value was high in 10 subgroups: retrospective, children, long-term iEEG, threshold (FRs and ripples) and automated detection and interictal (FRs). FSO after complete resection of FRs-area (crFRs-area) was achieved less often in people with TLE than extra-TLE (OR 0.37, 0.15–0.89, *p* = 0.006). Individual patient analyses showed that crFRs-area was seen more in patients with FSO with than without MRI lesions (*p* = 0.02 after multiple correction).

**Discussion:**

Complete resection of the brain area with HFOs is associated with good postsurgical outcome. Its prognostic value holds, especially for FRs, for various subgroups. The use of HFOs for extra-TLE patients requires further evidence.

## Introduction

Intracranial EEG (iEEG)–guided epilepsy surgery can cure people from drug-resistant epilepsy.^1,2^ The premise of surgery is accurate localization and complete removal of epileptogenic brain tissue. The epileptogenic zone (EZ) refers to a theoretical brain region that is necessary and sufficient to initiate epileptic seizures. No diagnostic technique so far can localize the EZ on its own, and the EZ is estimated based on various predictive measures.^3^ For capturing electrographic biomarkers, intraoperative electrocorticography (ioECoG) and diagnostic intracranial monitoring, including long-term electrocorticography (ECoG) and stereo-EEG, each has its advantages and shortcomings. High-frequency oscillations (HFOs), subdivided into fast ripples (FRs, 250–500 Hz) and ripples (80–250 Hz), have been identified as promising iEEG signal biomarkers for epileptogenic tissue delineation.^4-7^ Both types of brain oscillations have limitations: ripples can be physiologic (predominantly in eloquent regions) and pathologic,^3^ while FRs are scarce in occurrence.^8^ The prognosis of seizure outcome after complete resection of the HFOs-area (crHFOs-area) is the most consistent measure to test the clinical value of HFOs.^9-12^

Several studies demonstrated that HFOs-area removal tends to show better outcome compared with the area with interictal epileptiform discharges (IEDs).^8-11^ Patients with recurrent seizures after epilepsy surgery showed widespread HFOs in preresection ECoG recordings and residual FRs in postresection ECoG recordings.^13,14^ Some iEEG studies do not show advantages for HFOs.^15,16^ A Cochrane review in 2017 including 2 prospective studies (11 participants), with low evidence quality, concluded that prognostic value of HFOs was unreliable.^17^ So far HFOs are rarely used for clinical decision-making, although some studies have prospectively analyzed HFOs in iEEG data and evaluated whether crHFOs-area was associated with postoperative seizure freedom.^8,15,18,19^ The noninferiority randomized controlled “HFO trial,” compared surgical guidance with HFOs and IEDs in the ioECoG to seizure outcome. This trial challenges using HFOs as biomarkers for epileptogenic tissue, particularly in temporal lobe epilepsy (TLE).^4^

A meta-analysis identified 13 observational cohort studies and verified FRs as electrographic biomarkers of the EZ with moderate diagnostic accuracy.^20^ Next to answering the overall question whether HFOs are good predictors for surgical outcome, we have to consider that diverse clinical variables may reflect different results between various studies. These variables, which were not investigated in previous meta-analyses, include the study design, age group, diagnostic type, HFO identification method, whether an HFO rate threshold was used, iEEG state, sex, affected hemisphere, MRI findings, surgery location, and underlying pathology. We performed a 3-step systematic review and meta-analysis of this literature to (1) summarize the clinical characteristics and recording techniques, (2) assess the overall prognostic value of crHFOs-area for surgical outcome, and (3) determine the influence of clinical variables on this prognostic value.

## Methods

### Search Strategy

The systematic review and meta-analysis were conducted in accordance with the Preferred Reporting Items for Systematic Reviews and Meta-Analysis guideline (eAppendix 1). PubMed, Embase, and Web of Science were searched systematically (up to October 27, 2022) using the following terms as keywords: “high-frequency oscillation,” “epilepsy,” “surgery,” “resect,” and multiple variants of these terms (details in eMethods). A full-text review was performed when the screened abstract was considered relevant by Z. Wang and J. Guo. A consensus meeting was held in case of disagreement. We removed duplicates and searched reference lists of included articles to ensure more comprehensive literature retrieval.

### Eligibility Criteria

Study selection criteria were as follows: (1) people with epilepsy who underwent resective surgery; (2) HFOs (FRs and/or ripples) recorded in intracranial EEG (ioECoG, stereo-EEG, and long-term ECoG); (3) follow-ups ≥6 months after surgery; (4) seizure outcomes classified by Engel or International League Against Epilepsy (ILAE) scales or classification into seizure-free vs recurrent seizures; (5) crHFOs-area was mentioned or HFOs-area resection proportions were supplied per patient; (6) randomized controlled trials (RCTs), cohort studies, and case series reports (retrospective and prospective) of more than 5 patients who underwent epilepsy surgery and in whom intracranial HFOs (80–600 Hz; as some studies defined HFOs using 80/100–500/600 Hz) were analyzed; and (7) written in English and full text available.

Exclusion criteria were as follows: (1) articles reporting on high gamma frequencies and very HFOs (>600 Hz); (2) unpublished data, nonhuman studies, conference abstracts, editorials, reviews, and studies in languages other than English; (3) studies focusing on nonresective surgery such as vagus nerve stimulation; and (4) HFOs detected by noninvasive techniques, such as scalp EEG and magnetoencephalography.

### Data Extraction

Publications that fulfilled eligibility criteria would undergo data extraction by Z. Wang and J. Guo independently, including the following: (1) Publication details (publication year, full title, first author, country of origin, enrollment period, study design). (2) Clinical characteristics (age at surgery, sex, MRI findings, affected hemisphere, diagnostic type, HFO identification method, whether an HFO rate threshold was used, iEEG state (ictal or interictal), surgery location, underlying pathology, duration of postsurgical follow-up). (3) HFO type (FRs and ripples). We did not differentiate between physiologic and epileptic HFOs. (4) Classification of completeness of HFOs-area resection and/or calculation of the HFOs-area removal proportion per patient. Patients without HFOs detected were labeled as crHFOs-area because there are no HFOs remaining. We preferred to prioritize the analysis of HFOs detected post resection in patients with ioECoG subgroups if available; otherwise, we focused on HFOs recorded before resection. (5) Postsurgical seizure outcomes (Engel, ILAE, and seizure-free status).

### Surgical Outcome Measure

Favorable surgical outcome (FSO) was defined as Engel class I a-d, ILAE class 1, or seizure-free status at ≥6 months after epilepsy surgery. Nonfavorable surgical outcome (non-FSO) was defined as Engel class II-IV, ILAE class 2–6, or seizure recurrence.

We divided the study patients of each study into 4 groups: (1) patients with crHFOs-area and FSO; (2) patients with crHFOs-area and non-FSO; (3) patients without crHFOs-area and FSO; (4) patients without crHFOs-area and non-FSO. This was conducted separately for FRs and ripples.

### Quality Assessment

For the quality assessment of nonrandomized studies, we used the Newcastle-Ottawa Scale (NOS).^21^ For RCTs, we used the Cochrane risk of bias tool to categorize the bias as high or low. To assess the risk of bias, Z. Wang and J. Guo conducted an independent assessment, and disagreements were settled by S. Hoogteijling.

### Data Analysis

We synthesized study-level data and conducted whole-group analysis separately for complete resection of FRs-area (crFRs-area) and ripples-area (crRs-area) to evaluate surgical outcomes. The following data analyses were performed: (1) we calculated the overall pooled proportion of patients attaining FSO after crHFOs-area, using a random-effects model—addressing the potential interstudy heterogeneity—which integrated the Freeman-Tukey double-arcsine transformation to stabilize proportions and the Der Simonian-Laird technique to estimate between-study variance. This approach yielded a pooled proportion along with Clopper-Pearson 95% CIs. (2) We conducted a 2 × 2 contingency analysis using the Cochran-Mantel-Haenszel procedure with a random-effects model to compare the associations between FSO with and without crHFOs-area. Outcomes were presented as odds ratios (ORs) with 95% CIs based on the binomial distribution. (3) We used bivariate hierarchical modeling to evaluate the predictive performance of crHFOs-area. We plotted the summary receiver operating characteristic (SROC) curve and calculated the pooled sensitivity, specificity, and area under the curve (AUC). The AUC value was defined between 0.5 and 0.75 as a weak predictive performance and between 0.76 and 0.92 as a good predictive performance.^22,23^

Next, we extracted study-level data from the included studies and assessed the prognostic value for the following 12 subgroups categorized as follows: study design (prospective and retrospective), age group (children [≤20 years] and adults [>20 years]), diagnostic type (ioECoG and long-term iEEG [stereo-EEG or long-term ECoG]), HFO identification method (visual and automated), whether an HFO rate threshold was used (threshold and nonthreshold), iEEG state (interictal and ictal recording). The aforementioned subanalyses were performed separately for FRs and ripples. We classified the combined prognostic value for each subgroup as high if the following criteria were met: (1) pooled FSO proportion after crHFOs-area of ≥80%; (2) a significant association (lower bound of the 95% CI > 1) between crHFOs-area and FSO compared with without crHFOs-area; and (3) good predictive performance (AUC value >0.75) of the crHFOs-area. Meeting none of the 3 criteria was classified as a low combined prognostic value.

We compared surgery location (TLE vs extra-TLE) stratified by diagnostic type (ioECoG and long-term iEEG) at study level through dichotomous variable meta-analysis. Clinical variables of individual patients attaining FSO included sex (female, male), affected hemisphere (left, right), surgery location (TLE, extra-TLE), MRI findings (lesional, nonlesional), and underlying pathology. These variables were compared between crHFOs-area and without crHFOs-area patients for FRs and ripples separately (in total 10 groups), with categorical variables evaluated using Pearson χ^2^ tests and continuity correction. We applied the Benjamini-Hochberg procedure to calculate false discovery rates (FDRs) to correct for the effect of multiple comparisons among the 10 groups.

Statistics were conducted using R studio version 4.1.0 (meta, metafor, and meta4diag package) and Stata version 16.0. We calculated ORs and 95% CIs of all dichotomous variables for study-level data and considered *p* < 0.05 as statistically significant. Interstudy heterogeneity was assessed using Cochran *Q* and *I*^2^ statistics, in which significant heterogeneity was defined at *p* < 0.10 and *I*^2^ >50%. Individual patient analyses were assessed with a significance threshold of *p* < 0.05 after FDR correction.

### Publication Bias and Sensitivity Analysis

The study effect and the extent of potential publication bias were assessed by visual inspection of funnel plots for the pooled proportion and dichotomous variables meta-analysis in the whole group. The Egger test was conducted as a quantitative assessment of the funnel plot symmetry. Sensitivity analysis was conducted through leave-one-out cross-validation to evaluate the stability of dichotomous variable meta-analysis results.

### Standard Protocol Approvals, Registrations, and Patient Consents

The study was registered in PROSPERO (CRD42022353768). Because this was a systematic review and meta-analysis of publications, ethical standards committee approval and patient informed consent were not applicable.

### Data Availability

This article and its supplementary files encompass all the original and analyzed data during the course of this study.

## Results

### Study Selection and Characteristic

**Figure 1:**
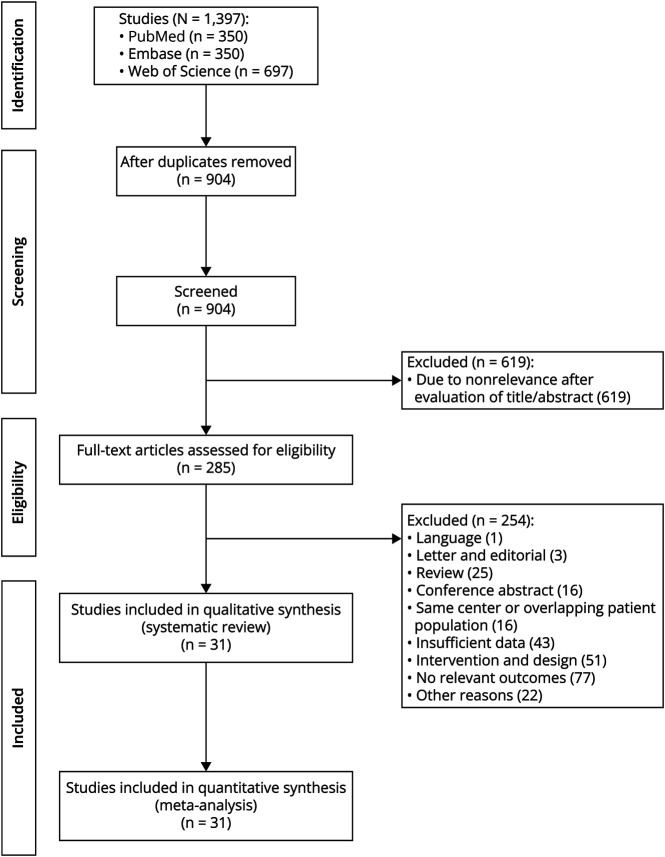
PRISMA Flowchart of Study Selection and Reasons for Exclusion PRISMA = Preferred Reporting Items for Systematic Reviews and Meta-Analysis.

The search yielded 1,387 references (Figure 1). After removal of 483 duplicates, the remaining 904 references were screened by title and abstract. We reviewed 285 full-text articles, resulting in the exclusion of 254. Finally, 31 original studies were included, which included 1 RCT, 7 prospective studies, and 23 retrospective studies, amassing 703 study participants (355 with crFRs-area, 247 without crFRs-area; 155 with crRs-area, 269 without crRs-area). Sixteen studies (411 patients) analyzed both FRs and ripples, 11 studies (257 patients) analyzed FRs only, and 4 studies (35 patients) analyzed ripples only. One hundred patients from 10 publications had complete resection of both FRs and ripple areas (Table 1). Two studies did not distinguish FRs and ripples but provided a generalized definition of complete resection of HFOs between 80 and 500 Hz.^9,24^ We assumed that FR and ripple areas were completely removed together and included these 2 publications into the FRs and ripple analysis groups. All included studies were observational cohort studies, except for 1 noninferiority RCT study^4^ and 1 nonrandomized trial without a control group.^25^

**Table 1:** Clinical Characteristics and Recording Techniques of the 31 Articles Included in the Meta-Analysis

Study (design)	Study center enrollment, year	Sample size	Age at surgery, y, median (range)	Diagnostic type	HFO identification method	Threshold (yes/no)	IEEG state	CrHFOs-area FRs/ripples/both No. of patients	Outcome parameter	Follow-up, mo, median (range)
#1 (P) Ramachandrannair et al.^25^	SickKids Canada; SC 2004–2005	5	10.0 (4.5–14)	Long-term ECoG	Visual	No	Ictal	NA/2/NA	Seizure-free	18.0 (12–29)
#2 (P) Modur et al.^12^	NNI USA; SC Not Stated	6	22.0 (19–32)	Long-term ECoG	Visual	No	Ictal	NA/4/NA	Engel	24.5 (20–38)
#3 (P) Hussain et al.^18^	MCH USA; SC 2008–2009	30	9.1 (1–20)	ioECoG	Visual	No	Interictal	28/NA/NA	Seizure-free	62.8 (17.2–86)
#4 (P) Fedele et al.^19^	USZ Switzerland; SC 2012–2016	20	40 (17–52)	Long-term ECoG	Automatic	Yes	Interictal SWS	14/11/9	ILAE	25 (10–46)
#5 (P) Jacobs et al.^15,a^	MCH USA; SC 2011–2012	20	9 (0.5–17)	ioECoG	Visual/automatic	Yes	Interictal SWS	16/16/16	Engel	12
#6 (P) Nariai et al.^8^	MCH USA; SC 2016–2018	14	14 (3–20)	Long-term ECoG	Visual	No	Interictal SWS	7/NA/NA	Seizure-free	24
#7 (P) Dimakopoulos et al.^37^	USZ Switzerland; SC 2015–2019	14	32 (18–53)	Long-term ECoG	Automatic	Yes	Interictal	3/3/NA	ILAE	27 (12–55)
#8 (RCT) Zweiphenning et al.^4^	UMC The Netherlands; SC 2014–2020	39	21 (12–39)	ioECoG	Visual	No	Interictal	29/29/29	Engel	12
#9 (R) Jacobs et al.^34^	MNI Canada; SC 2004–2008	20	42 (21–57)	Long-term ECoG	Visual	No	Interictal SWS	3/1/1	Engel	26 (13–37)
#10 (R) Wu et al.^40^	MCH USA; SC 2007–2008	24	8.5 (0.6–20)	ioECoG	Visual	No	Interictal	20/NA/NA	Seizure-free	26 (20–33)
#11 (R) Akiyama et al.^45^	SickKids Canada; SC 2004–2008	28	11 (1–18)	Long-term ECoG	Automatic	Yes	Interictal NREM	8/4/2	ILAE	24
#12 (R) Usui et al.^7^	NHSIEND Japan; SC 2005–2008	17	31 (16–43)	Long-term ECoG	Visual	No	Interictal/ictal	11/NA/NA	Engel	33 (12–54)
#13 (R) Fujiwara et al.^9,b^	CCHMC USA; SC 2008–2009	41	Mean 10 (0.8–25)	Long-term ECoG	Visual	No	Ictal	22/22/22	Seizure-free	Mean 14 (12–26)
#14 (R) Cho et al.^10^	SMC Korea; SC 2004–2009	15	30 (12–44)	Long-term ECoG	Automatic	Yes	Interictal SWS	6/4/3	Engel	26 (18–34)
#15 (R) Okanishi et al.^46^	SickKids Canada; SC 2006–2012	10	8.7 (2.7–18.4)	Long-term ECoG	Automatic	Yes	Interictal NREM	1/3/1	Engel	Mean 58 (19–76)
#16 (R) Fujiwara et al.^24,b^	CCHMC USA; SC 2008–2012	14	3.9 (2–16)	Long-term ECoG	Visual	No	Ictal	10/10/10	ILAE	Adequate time
#17 (R) Sakuraba et al.^6^	TUSM Japan; SC 2012–2014	12	26 (12–41)	Long-term ECoG	Visual/automatic	Yes	Interictal SWS	NA/2/NA	Engel	12
#18 (R) van't Klooster et al.^13^	UMC The Netherlands SC 2008–2012	54	15.5 (1–61)	ioECoG	Visual/automatic	No	Interictal	28/3/NA	Seizure-free	25 (17–38)
#19 (R) Wang et al.^20^	CCNI 2010–2011 SAHZU 2013–2014 Multi-Center	25	30 (12–59)	Long-term ECoG/stereo-EEG	Visual	Yes	Interictal SWS	5/10/NA	Engel	30 (23.3–35.9)
#20 (R) Feyissa et al.^28^	MCHR USA; SC 2016–2017	12	51 (18–68)	ioECoG	Visual/automatic	Yes	Interictal	NA/11/NA	Engel	8 (5–12)
#21 (R) Jiang et al.^43^	XWH China; SC 2016–2017	26	23 (5–42)	Long-term ECoG/stereo-EEG	Visual/automatic	Yes	Interictal SWS	9/2/NA	Engel	At least 12
#22 (R) Weiss et al.^30^	MCHR USA; SC 2012–2016	16	29 (14–50)	ioECoG	Visual/automatic	Yes	Interictal	12/NA/NA	Engel	18 (6–38)
#23 (R) Boran et al.^11^	USZ Switzerland; SC 2015–2018	22	20 (1–67)	ioECoG	Visual/automatic	Yes	Interictal	20/NA/NA	ILAE	29 (12–43)
#24 (R) Nevalainen et al.^38^	MNI Canada; SC 2010–2015	43	35 (14–55)	Stereo-EEG	Visual/automatic	Yes	Interictal SWS	10/NA/NA	Engel	At least 12
#25 (R) Qi et al.^5^	BTHNC China; SC 2015–2016	19	24 (3–33)	Stereo-EEG	Visual/automatic	Yes	Interictal/ictal	17/NA/NA	ILAE	Mean 10 (6–15)
#26 (R) Li et al.^44^	XWH China; SC 2016–2019	15	26 (13–42)	Long-term ECoG/stereo-EEG	Automatic	Yes	Interictal SWS	7/NA/NA	Engel	At least 12
#27 (R) van Klink et al.^14^	UMC The Netherlands; SC 2008–2015	41	Mean 16.6 (0.8–50)	ioECoG	Visual/automatic	No	Interictal	38/NA/NA	Engel	At least 12
#28 (R) Yu et al.^33^	TVGH China; SC 2012–2018	44	28 (10–66)	Long-term ECoG/stereo-EEG/ioECoG	Visual	No	Ictal/interictal	4/3/NA	Seizure-free	58.3 (24–214)
#29 (R) Weiss et al.^32^	TJU USA; SC 2016–2018	16	34 (20–47)	Stereo-EEG	Visual/automatic	Yes	Interictal	4/NA/NA	Engel	≥12
#30 (R) Bushara et al.^27^	NMH USA; SC 2015–2019	17	Mean 50.9 (20–79)	ioECOG	Visual	No	Interictal	2/8/NA	Seizure-free	Adequate time
#31 (R) Maccabeo et al.^e1^	UMC The Netherlands; SC 2008–2017	24	16 (3–48)	ioECOG	Visual	No	Interictal	21/7/7	Engel	≥12

Affiliations: BTHNC = Beijing Tiantan Hospital Neurosurgery Center; CCHMC = Cincinnati Children's Hospital Medical Center; CCNI = Cleveland Clinic Neurological Institute; MCH = Mattel Children's Hospital; MCHR = Mayo Clinic Hospital in Rochester; MNI = Montreal Neurologic Institute; NHSIEND = National Hospital Shizuoka Institute of Epilepsy and Neurological Disorders; NMH = Northwestern Memorial Hospital; NNI = Norton Neuroscience Institute; SAHZU = The Second Affiliated Hospital Zhejiang University School of Medicine; SickKids = The Hospital for Sick Children; SMC = Samsung Medical Center; TJU = Thomas Jefferson University; TUSM = Tohoku University School of Medicine; TVGH = Taipei Veterans General Hospital; UMC = Utrecht Medical Center; USZ = University Hospital Zurich; XWH = Xuanwu Hospital of Capital Medical University.

Abbreviations: CrHFOs-area = complete resection of HFOs-area; ECoG = electrocorticography; FR = fast ripple; HFO = high-frequency oscillation; iEEG = intracranial EEG; ILEA = International League Against Epilepsy; ioECoG = intraoperative electrocorticography; NA = not available; NREM = non-REM; (P) = prospective; (R) = retrospective; RCT = randomized controlled trial; stereo-EEG = stereo-EEG; SWS = slow-wave sleep.

a Study #5: the author used a concept of “majority removal” of HFOs for this multiple-center study. We extracted data from PEP-UCLA only because they provided the data of CrHFOs-area.

b Study #13 and study #16: the authors used the definition of “HFOs” as activity >80 Hz, and ripples and FRs were not separately distinguished.

Eligible studies came from 18 study centers, all tertiary epilepsy surgery referral centers, and 7 countries: single-center studies came from the United States (n = 11, patients = 210; 30%), Netherlands (n = 4, patients = 158; 22%), China (n = 4, patients = 104; 15%), Canada (n = 5, patients = 106; 15%), Korea (n = 1, patients = 15; 2%), Japan (n = 2, patients = 29; 4%), and Switzerland (n = 3, patients = 56; 8%). One multicenter study, who enrolled 25 patients (4%) from 2 countries (United States and China), was included.^26^ We extracted 1 cohort of ioECoG data from a multicenter study by Jacobs et al.,^15^ as the reported “majority removal” of HFOs in the other 2 cohorts (long-term iEEG) did not match our inclusion criteria. The main topics and purposes of the 31 included publications varied; 25 (80%) publications compared HFOs in iEEG with surgical outcomes; 13 (42%) publications compared the HFO distribution with that of IEDs for identifying the EZ; 6 (20%) publications focused on identifying the EZ in specific pathologies (tumors [3],^27-29^ tuberous sclerosis [2]^9,26^).

### Clinical Characteristics

Among the included 31 studies, 6 studies^27,29-33^ did not list individual patient characteristics. We requested data from corresponding authors or extracted relative data from figures and tables. Table 1 summarizes clinical characteristics and recording techniques of all included studies.

Eight studies (FRs in 140 patients; ripples in 77 patients) described crHFOs-area and surgical seizure outcome in children (median 10.5 years [range 0.5–20]) and 9 studies (FRs in 129 patients; ripples in 108 patients) in adults (median 30 years [range 21–79]). Thirteen patients from 2 studies had follow-up durations between 6 and 12 months,^28,32^ with a mean duration of 8 months. Twenty-seven studies followed up patients for ≥12 months after surgery, and 10 of these followed up patients for ≥24 months. Two studies stated “adequate time” for follow-up,^9,27^ rather than providing precise durations. The median follow-up time was 25 months (range 6–214 months) in the FR analysis group and 18 months (8–76 months) in the ripple analysis group. Seventeen (55%) of these studies reported postsurgical seizure outcomes using Engel classification; 8 (26%) studies used the seizure-free or not; and 6 (19%) studies used the ILAE classification of postsurgical seizure outcomes. On average, 64% of all included patients achieved postoperative favorable outcomes, with rates ranging from 5% to 84% per study. Notably, 1 study reported 1 seizure-free patient of 20 patients.^34^

### Whole-Group Meta-Analysis

#### The Pooled FSO Proportion

**Figure 2:**
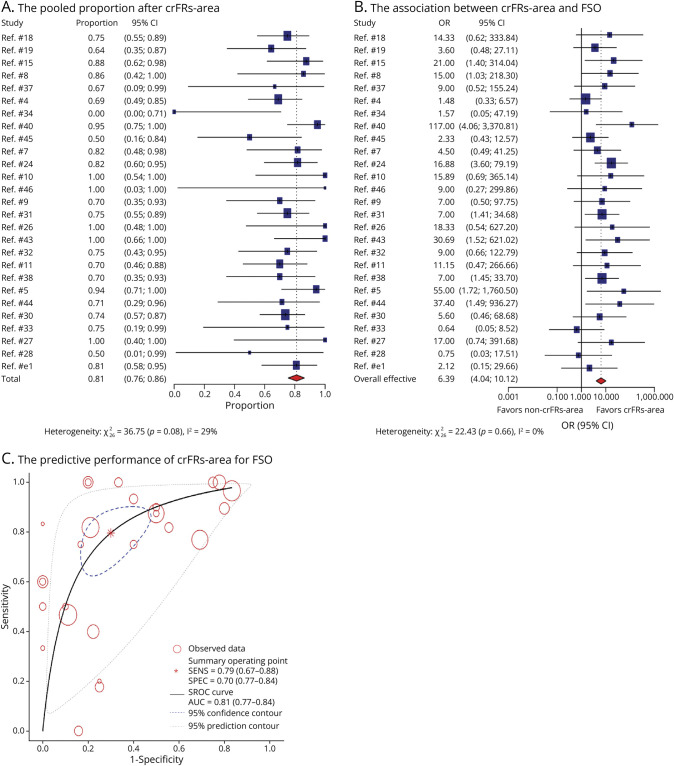
Whole-Group Meta-Analysis Results of the Prognostic Value of crFRs-Area for FSO The pooled FSO proportion in patients with crFRs-area (A). The comparison of crFRs-area and FSO with without crFRs-area and FSO (B). Each included study is represented by a blue square; the size of the square represents the relative importance of each study, with larger squares indicating studies carry greater statistical weight on the overall results. Each horizontal line put onto a forest plot represents a separate study being analyzed. And the pooled data with 95% CI is shown by a red diamond. The dotted vertical line represents the “line of null effect.” The predictive performance of crFRs-area for FSO (C). The summary operating point is surrounded by a blue dashed line representing the 95% confidence contour and a gray dotted line representing the 95% prediction contour. crFRs-area = complete resection of the fast ripple area; FSO = favorable surgical outcome.

There were 27 studies including 355 patients who underwent crFRs-area resection, among which 275 patients achieved FSO. The overall pooled FSO proportion of patients with crFRs-area was 81% (95% CI 76%–86%, *p* < 0.001, Figure 2A). There was low heterogeneity for the FR analysis group (*I*^2^ = 29%, *p* = 0.08).

**Figure 3:**
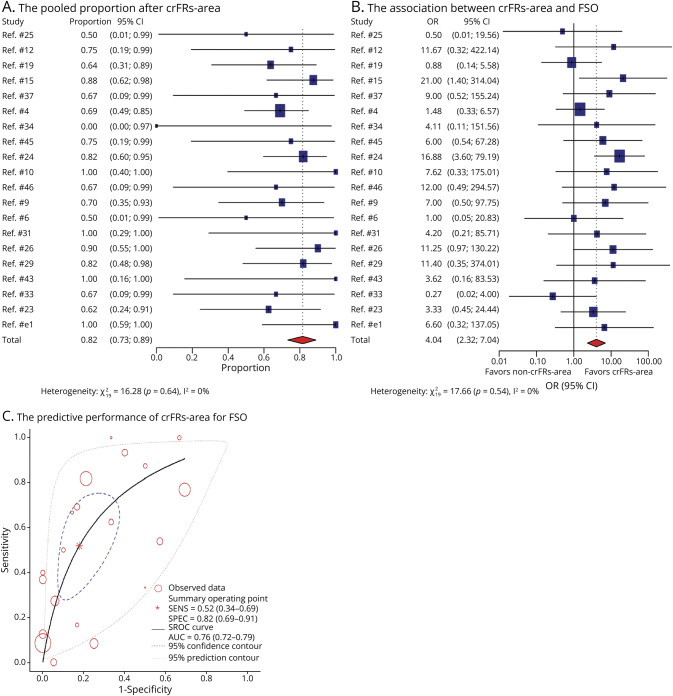
Whole-Group Meta-Analysis Results of the Prognostic Value of crRs-Area for FSO The pooled FSO proportion in patients with crRs-area (A). The comparison of crRs-area and FSO with without crRs-area and FSO (B). Each included study is represented by a blue square; the size of the square represents the relative importance of each study, with larger squares indicating studies carry greater statistical weight on the overall results. Each horizontal line put onto a forest plot represents a separate study being analyzed. And the pooled data with 95% CI is shown by a red diamond. The dotted vertical line represents the “line of null effect.” The predictive performance of crRs-area for FSO (C). The summary operating point is surrounded by a blue dashed line representing the 95% confidence contour and a gray dotted line representing the 95% prediction contour. crRs-area = complete resection of the ripple area; FSO = favorable surgical outcome.

There were 20 publications including 155 participants who underwent crRs-area, and 119 of them attained FSO. The pooled FSO proportion of patients with crRs-area was 82% (95% CI 73%–89%, *p* < 0.001, Figure 3A). There was low heterogeneity for the ripple analysis group (*I*^2^ = 0%, *p* = 0.64).

#### Comparison of crHFOs-Area With Without crHFOs-Area and FSOs

The likelihood of FSO was higher for crHFOs-area compared with without crHFOs-area (FRs OR 6.38, 95% CI 4.03–10.09, *p* < 0.001, Figure 2B; ripples OR 4.04, 95% CI 2.32–7.04, *p* < 0.001, Figure 3B). Pooling all results in the meta-analysis for FRs and ripples yielded a positive difference without any overlap with 1 on x-coordinate. The random-effects model yielded low heterogeneity for FRs and ripples with *I*^2^ = 0%, *p* = 0.67 and *I*^2^ = 0.4%, *p* = 0.55.

#### Predictive Performance of crHFOs-Area for FSO

For the FR analysis group, the pooled sensitivity and specificity were 0.79 (95% CI 0.67–0.88) and 0.70 (95% CI 0.57–0.80). The overall weighted AUC for crHFOs-area was 0.81 (95% CI 0.77–0.84), indicating good predictive performance. Significant heterogeneity was found for sensitivity (*I*^2^ = 81.97, 95% CI 74.80–89.15) and specificity (*I*^2^ = 65.10, 95% CI 48.55–81.64).

In the ripple analysis group, the pooled sensitivity was 0.52 (95% CI 0.34–0.69), and the pooled specificity was 0.82 (95% CI 0.69–0.91). The SROC curve had an overall weighted AUC value of 0.76 (95% CI 0.72–0.79), showing marginally good predictive performance. Significant heterogeneity was also found for sensitivity (*I*^2^ = 78.24, 95% CI 70.42–86.06) and specificity (*I*^2^ = 67.06, 95% CI 53.84–80.28).

### Subgroup Meta-Analyses

#### Fast Ripples

**Figure 4:**
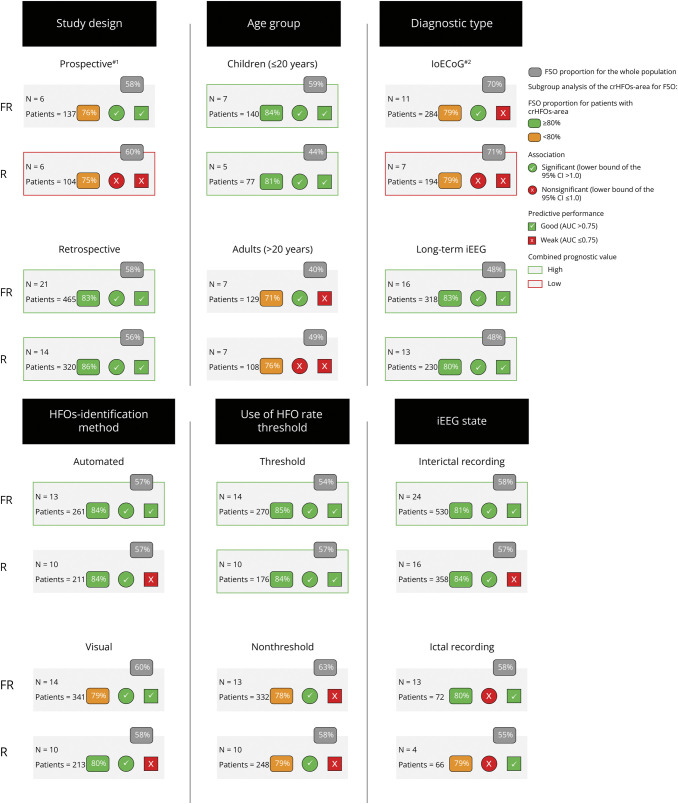
Subgroup Meta-Analysis Results of the Combined Prognostic Value of crHFOs-Area for FSO in Predefined Subgroups #1: The only RCT study of Zweiphenning et al. 2022 (study #8) was included in the prospective subgroup. #2: The UCLA study ioECoG cohort from the study of Jacobs et al. 2018 (study #5; multi-center study) was included in the ioECoG subgroup. AUC = area under the curve; crHFOs-area = complete resection of the high-frequency oscillations area; FR = fast ripple; FSO = favorable surgical outcome; HFO = high-frequency oscillations; iEEG = intracranial EEG; ioECoG = intraoperative electrocorticography; OR = odd ratio; R = ripples.

The pooled FSO proportion in patients with crFRs-area was <80% in 5 predefined subgroups: prospective studies, adults, ioECoG, visual detection, and nonthreshold. The proportion was ≥80% in the other 7 subgroups (retrospective, children, long-term iEEG, automatic detection, threshold, ictal, and interictal). CrFRs-area showed no association with FSO for the ictal recording subgroup and significant association for the other 11 subgroups. The SROC curves showed weak predictive performance of crFRs-area for FSO in 3 subgroups: ioECoG, adults; no HFO-rate thresholding and good predictive performance in the other 9 subgroups (Figure 4). For details of the subgroup analysis for FRs, see eTable 1.

#### Ripples

The pooled FSO proportion in patients with crRs-area is <80% in 3 predefined subgroups: prospective, adults, and nonthreshold; the proportion is ≥80% in the other 9 subgroups. CrRs-area was not associated with FSO for 3 subgroups (prospective, IoECoG, and ictal recording) and significantly associated in the other 9 subgroups. The SROC curves showed weak predictive performance of crRs-area for FSO in 7 subgroups: prospective, adults, IoECoG, visual, automatic, nonthreshold, and interictal recording and good predictive performance in the other 5 subgroups (Figure 4). For details of the subgroup analysis for ripples, see eTable 2.

#### Combined Prognostic Value of crHFOs-Area for FSO

CrHFOs-area showed a high combined prognostic value for FSO among 10 subgroups: retrospective (FRs and ripples), children (FRs and ripples), long-term iEEG (FRs and ripples), automated (FRs), threshold (FRs and ripples), and interictal recording (FRs). CrHFOs-area showed low value in 2 subgroups: prospective (ripples) and ioECoG (ripples).

### Meta-Analyses for Surgical Location in Patients With crHFOs-Area

**Figure 5:**
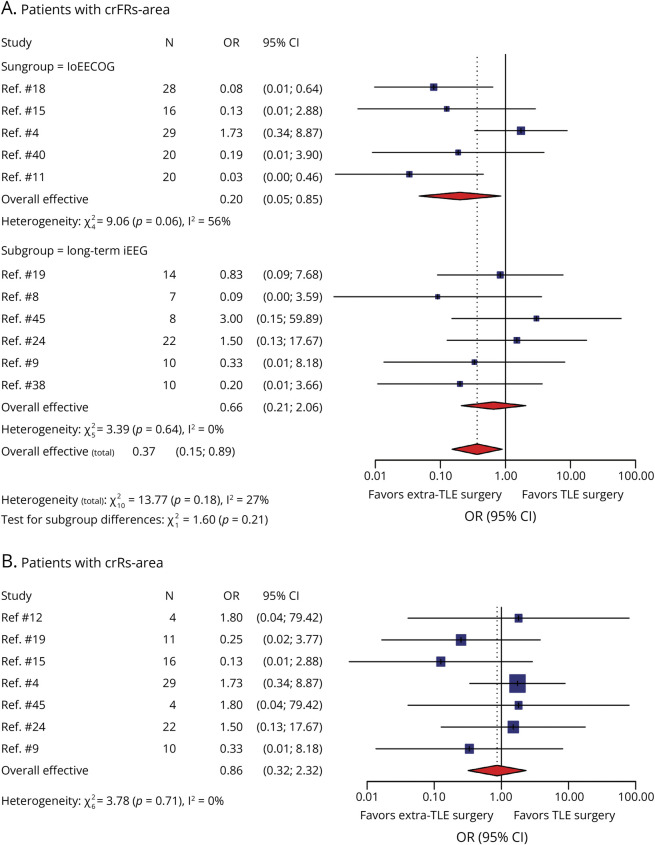
Meta-Analysis for Surgical Location in Patients With crHFOs-Area Forest plot of crFRs-area showed higher likelihood for extra-TLE surgery compared with TLE surgery for the whole group and ioECoG subgroup; there was no significant interdifference following subgroup of long-term iEEG (A). Forest plot of crRs-area showed no significant interdifference between extra-TLE surgery and TLE surgery group (B). An independent study is represented by each blue square; the size of the square represents the relative importance of each study, with larger squares indicating studies carry greater statistical weight on the overall results. Each horizontal line put onto a forest plot represents a separate study being analyzed. And the pooled data with 95% CIs are shown by red diamonds. The dotted vertical line represents the “line of null effect.” crHFOs-area = complete resection of the high-frequency oscillation area; crRs-area = complete resection of ripple area; crFRs-area = complete resection of fast ripple area; FSO = favorable surgical outcome; iEEG = intracranial EEG; ioECoG = intraoperative electrocorticography; TLE = temporal lobe epilepsy; N = total number of patients with crHFOs-area included.

We summarized surgical location in patients with crFRs-area from 11 studies, including 64 patients with TLE and 124 patients with extra-TLE. FSO was achieved less often in TLE compared with extra-TLE for the whole group (OR 0.37; 95% CI 0.15–0.89, *p* = 0.006) and the ioECoG subgroup (OR 0.20; 95% CI 0.05–0.85, *p* = 0.01, Figure 5A). There was low interstudy heterogeneity for the whole group (*I*^2^ = 27%, *p* = 0.18) and significant heterogeneity for the ioECoG subgroup (*I*^2^ = 56%, *p* = 0.06). In the subgroup of long-term iEEG, 6 studies including 71 patients compared TLE with extra-TLE. There were no between-group differences (TLE vs extra-TLE; OR 0.66; 95% CI 0.21–2.06; *p* = 0.35). Low interstudy heterogeneity was found for the subgroup of long-term iEEG (*I*^2^ = 0%, *p* = 0.64).

We extracted 99 patients with crRs-area from 7 studies, including 38 TLE and 61 extra-TLE. There was no between-group difference (TLE vs extra-TLE; OR 0.86; 95% CI 0.32–2.32, *p* = 0.65; Figure 5B). Low overall interstudy heterogeneity was found for the whole group (*I*^2^ = 0%, *p* = 0.71). There were insufficient study-level data to perform subgroup analysis of iEEG diagnostic type.

### Variable Analyses With Individual Patient Data

Using individual patient data with FSO, clinical variables between crHFOs-area and without crHFOs-area groups were compared in 10 groups (Table 2). From available data of FR analysis group, 93 patients with TLE surgery and 107 patients with eTLE surgery achieved FSO; In patients with FSO, crFRs-area seemed to appear more often in the extra-TLE surgery group than in the TLE surgery group (*p* = 0.04). This result was not significant after correction for multiple comparison (*p* FDR adjusted = 0.19). One hundred fifty-six patients with lesional MRI findings attained FSO, and 12 patients with nonlesional MRI attained FSO. In patients with FSO, CrFRs-area was observed more frequently in lesional MRI group compared with nonlesional group (*p* = 0.002; *p* FDR adjusted = 0.02). We did not find such statistical difference in ripple analysis group (surgical location *p* = 0.14, *p* FDR adjusted = 0.36; MRI findings *p* = 0.16, *p* FDR adjusted = 0.36).

**Table 2:** Clinical Characteristics of Individual Patients Attaining Favorable Surgical Outcome, Stratified by Sex, Affected Hemisphere, Surgery Locations, MRI Findings, and Pathologies

Variable	FR cohort, no. of studies (no. of patients)	No. of FSO		χ^2^ (*p* value)	*p* Value (FDR adjusted)	Ripples cohort, no. of studies (no. of patients)	No. of FSO		χ^2^ (*p* value)	*p* Value (FDR adjusted)
		CrFRs-area	Without CrFRs-area				CrRs-area	Without CrRs-area		
Sex	13 (263)			**1.32 (0.25)**	**0.42**	12 (210)			**0.63 (0.43)**	**0.54**
Male	13 (134)	68	16			12 (110)	36	32		
Female	13 (129)	76	11			12 (100)	40	27		
Affected hemisphere	**14 (335)**			**0.74 (0.39)**	**0.54**	**14 (373)**			**0.001 (0.98)**	**0.98**
Left side	14 (157)	64	20			14 (115)	37	31		
Right side	14 (178)	87	20			14 (258)	41	34		
Surgery location	**15 (340)**			**4.30 (0.04)** ^a^	**0.19**	**14 (249)**			**2.13 (0.14)**	**0.36**
TLE surgery	15 (169)	67	26			14 (134)	39	40		
Extra-TLE surgery	13 (171)	90	17			13 (115)	40	25		
MRI findings	**14 (289)** ^b^			**9.261 (0.002)** ^a^	**0.02** ^a^	**14 (263)**			**2.01 (0.16)**	**0.36**
Lesional	14 (262)	129	27			14 (232)	75	60		
Nonlesional	8 (27)	5	7			8 (31)	5	9		
Pathology	**16 (354)**			**2.89 (0.72)**	**0.80**	**15 (258)**			**7.62 (0.18)**	**0.36**
Tumor	11 (46)	33	5			9 (44)	21	12		
FCD	13 (118)	57	13			11 (68)	18	25		
Mesial temporal sclerosis	7 (31)	15	5			5 (17)	5	7		
Tuberous sclerosis	8 (41)	12	5			9 (38)	11	7		
Gliosis	7 (28)	8	2			8 (23)	8	2		
Others	13 (85)	31	10			11 (68)	17	15		

Abbreviations: CrHFOs-area = complete resection of HFOs-area; CrFRs-area = complete resection of fast ripple area; CrRs-area = complete resection of ripple area; FCD = focal cortical dysplasia; FDR = false discovery rate; FR = fast ripple; FSO = favorable surgical outcome; HFO = high-frequency oscillation; TLE = temporal lobe epilepsy.

a *p* < 0.05 was considered significant.

b Continuity correction was used for χ^2^ tests because the minimum expected count is 2.43.

Patient sex (FRs, *p* = 0.25; ripples, *p* = 0.43), affected hemisphere (FRs, *p* = 0.39; ripples, *p* = 0.98), and underlying pathology (FRs, *p* = 0.72; ripples, *p* = 0.18) did not show statistically significant differences between the crHFOs-area and without crHFOs-area groups (all *p* FDR adjusted >0.05).

### Quality of Assessment

eTable 3 summarizes that the NOS scores ranged from 7 to 9 stars, and the mean total score was 8.4 stars, considered as good quality on average. The RCT study showed a low risk of bias in terms of selection, performance, detection, attrition, reporting, and others (eTable 4).

### Bias Analysis and Sensitivity Analysis

Funnel plots of the dichotomous variables and pooled proportion meta-analysis in the whole group showed no asymmetry (Egger tests *p* > 0.05 in all groups); No group had obvious publication bias (eFigure 1 and 2).

Sensitivity analysis of the dichotomous variables yielded similar results in the whole group analysis (eFigure 3) and among 20 predefined subgroups (eFigure 4–9). In 4 additional subgroups (prospective ripples; ioECoG ripples; ictal FRs, and ripples), the results of the sensitivity analysis did not match those of the primary studies (eFigure 4, 6, and 9).

## Discussion

Barriers to consider HFOs in iEEG recordings for actual surgical decision-making include ethical and practical issues, accompanied by incongruent studies with relatively small sample sizes. This comprehensive systematic review and meta-analysis of 31 publications from 18 tertiary epilepsy centers, amassing more than 700 patients makes several key contributions to assess the prognostic value of removing the whole brain area with HFOs for FSO in patients with epilepsy surgery. First, the complete resection of FR and ripple areas give invariantly similar pooled FSO proportions (81% for FRs; 82% for ripples). Second, we demonstrated that while complete resection of both FR and ripple areas significantly contributed to FSO, FRs performed as more accurate biomarkers of FSO than ripples. Third, our subanalyses for various clinical factors validated the high combined prognostic value of complete resection of FRs and/or ripples in 10 of 24 predefined subgroups; this value was absent for ripples in prospective study designs and ioECoG studies. Fourth, our study-level results suggest that in patients with complete FRs-area removal, ioECoG-guided tailoring proved superior to achieve FSO in extra-TLE compared with TLE. Fifth, the individual patient analysis showed that complete FRs-area removal occurred most often if MRI lesions were present. Finally, the robustness of our results was supported and explained through publications bias assessment and sensitivity analysis.

Controversy surrounds the question which type of HFOs is better to delineate the margins of the surgical resection. FRs have been suggested to be more pathologic and more strongly linked to epileptogenicity than ripples.^31^ An earlier meta-analysis conducted by Höller et al.^35^ in 2015 showed a higher resection ratio of both types of HFOs in seizure-free patients compared with seizure-recurrent patients. This is in line with our findings. The SROC plots revealed lower but not statistically different predictive performance of ripples. The existence of physiologic ripples is the main flaw for ripples as a prognostic biomarker.^36^ Hussain et al.^18^ showed in 60 patients that residual FRs independently induce a greater than 25-fold increased risk of seizures. FRs and ripples were analyzed separately in this study. The scattered and small sample size limited analysis for complete resection of simultaneous occurring FR and ripple areas at study level. The combination of FRs and ripples may be more enabling than either alone.^19,37^

The prognostic value of complete resection of HFOs-area differed among the 24 predefined subgroups. Most included studies were observational retrospective studies. Besides the “HFO trial,” we included 7 “prospective studies” in which clinicians were blinded to the marked HFO results and postoperative seizure outcomes. We found a higher pooled FSO proportion for FRs and ripples in retrospective than prospective studies. None of these “prospective studies” used HFOs for surgical planning, which means that a causal link between HFO resection and postoperative outcomes could not be explored. We found that complete ripple area removal in prospective and ioECoG subgroups had low combined prognostic value. Conversely, complete HFOs-area (FRs and ripples) removal in retrospective and long-term iEEG subgroups showed high association and predictive performance for FSO, as previously reported.^8,38^ Future large-size prospective studies could clarify whether the low value observed in ripple analysis is genuine or influenced by the small cohort. In addition, direct comparisons of ioECoG, stereo-EEG, and long-term ECoG are needed.

The “HFO trial” did not show noninferiority of HFOs compared with IEDs for the whole group but showed noninferiority for the subgroup of extra-TLE after confounder correction.^4^ In this study, we explored the effect surgery location at study level and patient level. The study-level analysis showed that patients with extra-TLE surgery who underwent complete resection of the area showing FRs were more likely to achieve FSO compared with those who underwent TLE surgery, for the whole group and the ioECoG subgroup, but not for the long-term iEEG subgroup. We extracted patients with complete FRs-area removal from the “HFO trial” (29 of 39 patients),^4^ which showed an odds ratio opposite to the other 4 ioECoG studies^8^ (OR 1.73; Figure 5), yielding significant heterogeneity.^4^ Our patient-level analysis indicated that complete FRs-area removal in patients with FSO seemed more frequent in the extra-TLE group than in the TLE group; this trend did not survive FDR correction. Multiple comparison correction can be conservative in small sample sizes, making it challenging to detect significant differences. The current findings supporting the use of HFOs for extra-TLE patients seems relatively weak and requires further studies that include direct comparisons and large sample sizes.

We observed a high combined prognostic value of complete HFOs-area removal for FSO in children vs adults. Commonly, children with complete removal of MRI lesions are more prone to achieve FSO than adults.^39^ This phenomenon can be partly explained by a typically larger resected volume in children than in adults, increasing the chance on good outcome. HFOs predicted surgical outcomes better in the group using ioECoG recordings in children than in the long-term iEEG recordings in adults.^40^ The epileptogenic tissue is often close to the cortical surface in the pediatric population, which enables detecting HFOs-area with grids/strips and even scalp EEG.^41^ Age-related changes seem to influence HFOs, which needs a next step to assess across childhood and adolescence.^42^

HFO rate thresholding is vital when presenting HFO results. Our subanalyses identified a high combined prognostic value of complete resection of FR and ripple areas in HFO rate threshold subgroup, only if an HFO rate threshold was applied. Jiang et al.^43^ and Li et al.^44^ set up a quantitative threshold (HFO rate >1 event per minute). Thresholding can also be established using statistical guideline, including 95% HFO rate distributions,^19,37^ statistical dispersion (e.g., Tukey Fence),^10,15^ and standard deviation (e.g., Kittler method).^45,46^ Uniform methods are needed to define the threshold for HFOs algorithms to enable direct statistical comparisons across studies.

The EZ remains theoretical due to uncertainties; seizure freedom after surgery indicates the entire EZ is removed, but does not reflect its size. MRI lesions enable an improvement in preoperative localization of the epileptogenic focus.^47^ The absence of MRI-visible lesions has been identified as a key predictor of surgical failure. These cases require a more comprehensive presurgical examination including intracranial monitoring.^48^ Our individual analysis findings showing that people with MRI lesions had most often complete FRs-area removal were in accordance with earlier studies.^48-50^ This result was limited by the small nonlesional MRI sample size.

Our study has limitations. The foremost methodologic weakness is the varying definition of complete resection across studies, which should be considered when interpreting the results because it may signify incomplete HFOs-area removal in certain cases. Second, FRs and ripples were mixed in several included publications. There were 100 patients from 10 publications who underwent complete resection of combined FR and ripple areas. The repeat inclusion might have led to an indistinguishable effect, increasing the weight of these studies into both FR and ripple analyses. Third, iEEG HFO recording and analysis methodology is not standardized and the inter-reviewer reliability is often inadequate.^e4^ HFO characteristics, including duration, amplitude, and peak frequency vary, which means that patients categorized as having complete HFOs-area removal in 1 center may have residual HFOs signal in another center. There should be a push for standardization of HFOs-recording and analysis to improve study outcome comparison. Fourth, we must consider physiologic and epileptic HFO overlap in frequency. Physiologic HFOs are not limited to mesial temporal structures and occur in nonepileptic functionally eloquent regions such as the occipital lobes and perirolandic region.^e3,e4^

Our statistical findings should not be mistaken for the implication that resection of every individual electrode site exhibiting HFO is necessary or sufficient to achieve FSO; An all-purpose interpretation for the prognostic value of HFOs may not exist, and further studies are required to investigate the value of this biomarker in a case-by-case fashion. By searching ClinicalTrials.gov, we discovered 2 ongoing interventional clinical trials: 1 double-blinded RCT assessing whether HFO-generating tailored surgery can lead to better seizure outcomes (NCT03790280; n = 30); another small interventional clinical trial focusing on the functional utility of stereotyped HFOs with the Brain Interchange implantable system (NCT05439655; n = 20). Future multi-institutional collaborations collating individual patient data are required to investigate factors that could not be assessed in our analysis, including specific surgery location and more pathologic categories.

## Appendix Authors

**Table:**

Name	Location	Contribution
Ziyi Wang, MD	Department of Neurology and Neurosurgery, University Medical Center Utrecht Brain Center, University Medical Center Utrecht, Part of ERN EpiCARE, the Netherlands	Drafting/revision of the article for content, including medical writing for content; major role in the acquisition of data; study concept or design; analysis or interpretation of data; and additional contributions: quality assessment of the included studies
Jiaojiao Guo, MD	Department of Neurology and Neurosurgery, University Medical Center Utrecht Brain Center, University Medical Center Utrecht, Part of ERN EpiCARE, the Netherlands	Drafting/revision of the article for content, including medical writing for content; major role in the acquisition of data; analysis or interpretation of data; and additional contributions: quality assessment of the included studies
Maryse van 't Klooster, PhD	Department of Neurology and Neurosurgery, University Medical Center Utrecht Brain Center, University Medical Center Utrecht, Part of ERN EpiCARE, the Netherlands	Drafting/revision of the article for content, including medical writing for content; study concept or design
Sem Hoogteijling, PhD	Department of Neurology and Neurosurgery, University Medical Center Utrecht Brain Center, University Medical Center Utrecht, Part of ERN EpiCARE, the Netherlands	Drafting/revision of the article for content, including medical writing for content; analysis or interpretation of data; and additional contributions: quality assessment of the included studies
Julia Jacobs, PhD	Department of Pediatrics, University of Calgary, Alberta Children's Hospital, Calgary, Canada	Drafting/revision of the article for content, including medical writing for content
Maeike Zijlmans, MD	Department of Neurology and Neurosurgery, University Medical Center Utrecht Brain Center, University Medical Center Utrecht, Part of ERN EpiCARE; Stichting Epilepsie Instellingen Nederland (SEIN), Heemstede, the Netherlands	Drafting/revision of the article for content, including medical writing for content; study concept or design

## Glossary

AUC: area under the curve
crHFOs-area: complete resection of the HFOs-area
crFRs-area: complete resection of the fast ripple area
crRs-area: complete resection of the ripple area
ECoG: electrocorticography
EZ: epileptogenic zone
FCD: focal cortical dysplasia
FDR: false discovery rate
FR: fast ripple
FSO: favorable surgical outcome
non-FSO: nonfavorable surgical outcome
HFO: high-frequency oscillation
IED: interictal epileptiform discharge
iEEG: intracranial EEG
ioECoG: intraoperative ECoG
ILAE: International League Against Epilepsy
NOS: Newcastle-Ottawa Scale
OR: odds ratio
RCT: randomized controlled trial
SROC: summary receiver operating characteristic
TLE: temporal lobe epilepsy
